# Selenium (Se) and the risk of breast, ovarian and prostate cancers

**DOI:** 10.1186/1897-4287-10-S3-A8

**Published:** 2012-04-20

**Authors:** Katarzyna Jaworska, Anna Jakubowska, Katarzyna Durda, Tomasz Huzarski, Pablo Serrano-Fernandez, Grzegorz Sukiennicki, Magdalena Muszyńska, Tomasz Byrski, Jacek Gronwald, Satish Gupta, K Kaczmarek, Jan Lubiński

**Affiliations:** 1 Pomeranian Medical University and Read Gene SA, Szczecin, Poland

## Aim of the study

The aim of the study was determination of serum Se concentration and identification of genetic variations in genes related to metabolism of selenium as markers of cancer risks for carriers of BRCA1 gene mutation and individuals with susceptibility to other common unselected cancers (breast, ovarian, prostate).

## Material and methods

Eight genotypes of 4 most common SNPs localised in GPX1, GPX4, TXNRD2 and SEP15 were selected. Genotyping was performed in 93 affected and 186 unaffected matched BRCA1 carriers as well as on pairs matched 1:1 consisting of 108 breast, 50 ovarian and 105 prostate consecutive cancer patients and healthy controls.

The following techniques for laboratory analyses have been applied: a) sequencing on ABI310, b) SimpleProbe or TaqMan analysis (a melting-curve genotyping with fluorescence-labeled probes based on the LightCycler 480 System (Roche Applied Science), c) determination of selenium concentration in plasma using atomic absorption spectrometer AAnalyst600 (Perkin Elmer).

## Results

In none of studied groups statistically significant differences on cancer risk could be found when serum selenium concentration was assessed as a single factor. However, when selenium level data were combined with some selenoprotein genotypes some strong associations with cancer risk have been identified.

### BRCA1

The strongest association was found for carriers of SEP15 nGG genotype (Tab.1). Additionally, significantly lower risk of cancers were found for GPX1 CC (for Se level >80µg/l, ~ 6 times) and GPX4 CC (for Se level >100µg/l, ~10 times).

**Table 1:** Correlation between cancer risk and serum selenium concentration in carriers of SEP15 nGG genotype.

BRCA1 gene mutation carriers						
Genotype	Se	No		Chi^2^teste		
	
		Cancers	Controls	p	OR	CI

Sep15nGG	<56µg/l	16	6	0.0005	43	2.2-861
		
	>95µg/l	0	8			

Unselected breast cancers carriers						

Genotype	Se	No		Chi^2^teste		
	
		Cancers	Controls	p	OR	CI

Sep15nGG	<56µg/l	7	5	0.009	18.2	1.8-188
		
	>95µg/l	1	13			

### Unselected breast cancers

The strongest association was found for carriers of SEP15 nGG genotype (Tab.1).

Significantly lower risk of cancer was found also for TXNRD2 GG (for Se level 60 - 80µg/l, ~ 4 times) and GPX1 nCC/TXNRD2 nGG/SEP15 GG (for Se level <55µg/l, ~10 times).

### Unselected ovarian cancers

Results were not statistically significant however tendencies similar to those found in BRCA1 carriers and unselected breast cancer were observed.

### Unselected prostate cancers

For four genotypes: GPX1 CC, GPX4 nCC, TXNRD2 GG and SEP15 nGG lower risk (~3 times) was found for selenium level ~90µg/l.

